# Confirmation of Maslow’s Hypothesis of Synergy: Developing an Acceptance of Selfishness at the Workplace Scale

**DOI:** 10.3390/ijerph13050462

**Published:** 2016-04-30

**Authors:** Jiro Takaki, Toshiyo Taniguchi, Yasuhito Fujii

**Affiliations:** 1 Department of Public Health, Sanyo Gakuen University Graduate School of Nursing, 1-14-1 Hirai, Naka-ku, Okayama-shi, Okayama 703-8507, Japan; 2 Department of Welfare System and Health Science, Okayama Prefectural University, 111 Kuboki, Soja-shi, Okayama 719-1197, Japan; taniguti@fhw.oka-pu.ac.jp (T.T.); fujii@fhw.oka-pu.ac.jp (Y.F.)

**Keywords:** acceptance of selfishness, Abraham H. Maslow, altruism, calling, job control, mental health, Ricardo Semler, sense of contribution, synergy, work engagement

## Abstract

This study aimed to develop a new Acceptance of Selfishness at the Workplace Scale (ASWS) and to confirm Maslow’s hypothesis of synergy: if both a sense of contribution and acceptance of selfishness at the workplace are high, workers are psychologically healthy. In a cross-sectional study with employees of three Japanese companies, 656 workers answered a self-administered questionnaire on paper completely (response rate = 66.8%). Each questionnaire was submitted to us in a sealed envelope and analyzed. The ASWS indicated high internal consistency (Cronbach’s alpha = 0.86). Significant (*p* < 0.001) positive moderate correlations between ASWS scores and job control scores support the ASWS’s convergent and discriminant validity. Significant (*p* < 0.001) associations of ASWS scores with psychological distress and work engagement supported the ASWS’s criterion validity. In short, ASWS was a psychometrically satisfactory measure. Significant (*p* < 0.05) interactions between a sense of contribution and acceptance of selfishness at the workplace in linear regression models showed that when those two factors are low, psychological distress becomes high. However, when a sense of contribution and acceptance of selfishness are high, work engagement also becomes high. Thus, Maslow’s hypothesis of synergy was confirmed.

## 1. Introduction

Ruth Benedict has defined *synergy* as social-institutional arrangements that fuse selfishness and unselfishness by transcending their polarity so that the dichotomy between them is resolved, transcended and configured into a new, higher unity. This is to be arranged by institutions so that when one pursues selfish gratifications, one automatically helps others, and when one is altruistic, one automatically rewards and gratifies oneself [1]. Benedict suggested synergy as a function of developed culture [1]. Abraham H. Maslow, an American psychologist known for creating Maslow’s hierarchy of needs culminating in self-actualization, has suggested that this is similarly true within the individual; if selfishness and unselfishness are mutually exclusive, it signifies mild psychopathology, and synergic ability is almost synonymous with psychological health [1].

Research on Maslow’s hypothesis of synergy seems to be important. It can, for example, clarify the mechanisms of how social capital affects mental health. Social capital has been defined as norms of reciprocity and trust, civic participation, and formal and informal associations that facilitate collective action for mutual benefit [2]. Social capital is protective for mental health; various underlying mechanisms have been proposed to explain the association between social capital and mental health, for example social support, social networks and social control over health-risk behaviors [3]. However, these mechanisms have not been fully established [3]. When we consider social capital’s affinity with synergy, the essence of social capital’s effect on mental health can be synergy.

Ricardo Semler, a visiting scholar at Harvard and the Massachusetts Institute of Technology (MIT) with an Owner/President Management (OPM) degree from Harvard Business School [4], has created one of the world’s most successful and inspirational companies [5]. From 1994 to 2001, Semco, his company, increased its annual revenue from 35 million to 160 million US dollars [5]. Semco achieved unique market niches, has highly motivated employees, and has low turnover and diverse product and service areas [5]. Semco has relinquished control over its workers, so they can behave selfishly in the workplace as follows: After being taught how to read balance sheets and cash-flow statements through a labor union–developed course, they can determine their own salaries based on financial information disclosed by Semco [5,6]. They can follow their interests and instincts when choosing jobs and projects [5]. They can seek personal challenges and satisfaction before trying to meet the company’s goals [5]. They can question, analyze, investigate and dissent in the workplace [5]. They can have a free week to discover their true talents and interests [5]. They can merge their personal aspirations with the company’s goals [5]. Then, their efforts naturally translate into the company’s profit, growth and longevity [5]. In other words, they contribute to the company.

Semler suggested that ‘a calling’ is work worth doing, which agrees with the concept of goals in one’s life [5]. If a worker behaves selfishly and contributes to the company, in other words merges his/her personal aspirations with the company’s goals, his/her work should be a calling. Maslow’s synergy seems to equate to a calling. Scholarly interest in the concept of a calling is increasing. Wrzesniewski described a calling as ‘work as an end in itself with a belief that it contributes to the greater good’ [7]. People with ’a calling’ orientation find more meaning in their work [7]. Several researchers indicated that a calling is positively associated with work engagement [8,9,10]. McManus *et al.* suggested that ‘a calling’ is almost synonymous with work engagement [11]. Barak, Barush and their colleges indicated that ’a calling’ was not positively associated with life satisfaction among mental health professionals [12]. Duffy *et al.* suggested that ‘living a calling’ (actualizing one’s calling in one’s current career) was positively associated with work meaning, life meaning, job satisfaction and life satisfaction and that in a longitudinal study, career commitment, work meaning and job satisfaction seemed to be antecedent to ‘living a calling’ [13,14]. Research on Maslow’s hypothesis of synergy may accelerate understanding on how to live a calling.

If a worker behaves selfishly at the workplace and contributes to others, he/she should be synergic [1]. Based on Maslow’s hypothesis, such a person should be psychologically healthy [1]. In this study, we tried to confirm this hypothesis. The first problem we faced was how to measure selfishness. Should we measure the workers’ selfish behavior directly or measure the acceptance of selfishness at the workplace? We considered possible future intervention studies that may be inspired by this study. Elevating selfish behavior might be difficult because human behavior does not always change as expected; however, acceptance of selfishness at the workplace depends on labor regulations, which can be changed in each department. Health outcomes can be easily compared between workers in departments that allow selfishness at the workplace and those in departments that do not. Measurement of the latter seemed to be more useful. Thus, the first aim of this study was to develop a new scale of acceptance of selfishness at the workplace. The second aim of this study was to confirm that if both degrees of acceptance of selfishness at the workplace and a sense of contribution are high, the worker was synergic and psychologically healthy. We are unaware of any previous study confirming Maslow’s hypothesis of synergy.

## 2. Materials and Methods

### 2.1. Participants

This cross-sectional study’s participants were recruited from all the workers (*n* = 982) at a manufacturing company and two service companies in Japan. The organizations were selected because they were convenient to investigate. The purpose and procedure of the study’s survey were explained to the participants in documents. From May to September 2015, 674 workers submitted written informed consent for inclusion, answered a self-administered questionnaire on paper and submitted it to us in a sealed envelope. Among them, 18 (2.7%) had incomplete data. Azen *et al.* indicated that complete cases analyses and Expectation-Maximization (EM) imputations performed equally well regardless of the correlational structure when the percentage of incomplete data was only 5% [15]. Thus, we performed complete cases analyses (*n* = 656, final response rate = 66.8%). The study was conducted in accordance with the Declaration of Helsinki, and the protocol was approved by the Ethics Committee of Okayama Prefectural University (No. 464).

### 2.2. Measures

The self-administered questionnaire survey included demographic questions (age, sex, marital status, educational level, work status and lifestyles), the new scale for acceptance of selfishness at the workplace, and measures of job control, psychological distress, work engagement and sense of contribution. Categorical variables were coded as follows: males = 1, females = 0; married = 1, others = 0; educated for 12 years or more = 1, educated for less than 12 years = 0; supervisory or management position = 1, others = 0; exercising once per week or more = 1, exercising less than once per week = 0.

To determine items that would constitute the new Acceptance of Selfishness at the Workplace Scale (ASWS), we examined sentences in Maslow’s and Semler’s work, selecting from among them. Additionally, we included new items and drafted six items proposed to measure exhaustively and exclusively acceptance of selfishness at the workplace. The ASWS consists of these six items, measured on a five-point response option from 1 (strongly disagree) to 5 (strongly agree). The total score (ranging from 6 to 30) reflects the degree of acceptance of selfishness in the workplace. Both English and Japanese versions of the ASWS were prepared. Original items in English were translated into Japanese by a bilingual person and then back-translated into English by another bilingual person. A native English speaker then checked the back-translation. Translation and back-translation were repeated until no differences in meaning between the original and back-translated items were found. Items are shown in Table 1.

Job control (job-decision latitude) was measured using the Job Content Questionnaire (JCQ), developed by Karasek and based on the demands-control model [16]. The JCQ includes scales for job control (nine items; range, 24–96), with a four-point response option from 1 (strongly disagree) to 4 (strongly agree). Higher scores indicate greater job control [16]. The JCQ was translated into Japanese and its internal consistency reliability and factor and construct validity have been reported as acceptable [17].

The Kessler Psychological Distress Scale (K6), which consists of six items measured on a five-point scale (0–4), was used to evaluate psychological distress, with a total score ranging from 0 to 24. Higher scores indicate more severe psychological distress [18]. Translated into Japanese, the K6 has been shown to have acceptable internal reliability and validity for measuring DSM-IV (Diagnostic and Statistical Manual of Mental Disorders, Fourth Edition) mood and anxiety disorders, as assessed by diagnostic interviews administered by a lay interviewer in a sample community [19].

Work engagement was assessed using the short form of the Utrecht Work Engagement Scale (UWES), which has been validated in Japan [20,21]. The UWES includes three subscales (each consisting of three items): vigor, dedication and absorption. Vigor is characterized by high levels of energy, the willingness to invest effort in one’s work and persistence in the face of difficulties. Dedication refers to being strongly involved in one’s work and experiencing feelings of significance, enthusiasm, inspiration, pride and challenge. Absorption is characterized by being fully concentrated on and engrossed in one’s work so that time passes quickly, and there is difficulty in detaching oneself from one’s work [20]. All items are scored on a seven-point Likert-type scale, ranging from 0 (never) to 6 (always). Each total score (ranging from 0 to 18) also produces a score for each subscale. The total score of the three subscales (ranging from 0 to 54) is the score for work engagement [20]. Higher scores indicate greater work engagement, which has been used as a positive psychological health outcome [20,21].

Sense of contribution was assessed using the Sense of Contribution Scale (SCS) [22]. The SCS consists of seven items (e.g., I feel useful to my superiors in my workplace) measured on a four-point scale (1 = never; 2 = rarely; 3 = sometimes; 4 = frequently). The total score (ranging from 7 to 28) reflects one’s sense of contribution, with higher scores indicating a greater sense of contribution [22]. Both English and Japanese versions of the SCS were prepared using the back-translation method [22]. The SCS showed high internal consistency, test-retest reliability, and convergent, discriminant and criterion validity [22].

### 2.3. Statistical Analyses

Exploratory factor analysis was conducted on the ASWS; factors with eigenvalues greater than 1 were extracted, using the least-squares method and promax rotation to obtain the factor structure. To show exclusive measurement of ASWS items, we calculated Spearman’s correlations among them. Then we assessed the scale’s reliability and validity. Cronbach’s alpha coefficient was calculated to test its internal consistency reliability.

Job control is similar to but different from acceptance of selfishness at the workplace. For example, the JCQ job control items “My job allows me to make a lot of decisions on my own” and “I have an opportunity to develop my own special abilities” might overlap with acceptance of selfishness at the workplace. However, the JCQ job control item “My job requires that I learn new things” does not seem to do so [23]. Furthermore, ASWS item 1, “I can question freely at the workplace”, did not seem to fully overlap with job control. For actualization, the company must be flexible enough to listen to the employee’s question and answer it. Thus, this item seems to be involved in workplace support rather than job control. However, some ASWS items seem to overlap with job control, at least partially. Therefore, we used job control to assess convergent and discriminant validity. We hypothesized that acceptance of selfishness at the workplace has a moderate but not excessively high positive correlation with job control. Pearson’s correlations were assessed.

Maslow has suggested that the synergistic ability is almost synonymous with psychological health [1]. Selfishness is an aspect of synergy [1]. Thus, with regard to criterion validity, we hypothesized that acceptance of selfishness at the workplace was antecedent to psychological distress and work engagement, and that psychological distress was inversely associated and work engagement was positively associated with acceptance of selfishness at the workplace. Pearson’s correlations and multivariable linear regression analyses adjusted for potential confounders (age, sex, marital status, educational level, work status, exercise, job control and sense of contribution) were assessed.

After developing the ASWS (the first aim of this study), we conducted the second aim of this study, a confirmation of Maslow’s hypothesis of synergy [1], as follows:

1. When both a sense of contribution and acceptance of selfishness at the workplace are low, psychological distress becomes significantly high.
2. When both a sense of contribution and acceptance of selfishness at the workplace are high, work engagement becomes significantly high.

These interactions were assessed by hierarchical regression analyses. In model 1, the variables of sense of contribution and acceptance of selfishness at the workplace and the product of both variables were entered as independent variables in multivariable regression models with work engagement and psychological distress as dependent variables. In model 2, all the aforementioned covariates were added to independent variables to control for potential confounding effects. In accordance with Jaccard *et al.* [24], continuous variables used as independent variables were mean-centered. To examine this interaction further, graphic displays of regression models were also created based on recommendations of Cohen *et al.* [25]. Regression lines and predicted values illustrating significant interactions were constructed from intercepts, unstandardized regression coefficients, mean values and standard deviations (SDs). Scores were plotted at low (one SD below the mean) and high (one SD above the mean) values. Scores were calculated to an accuracy of six decimal points.

All *p*-values were two-tailed, and *p* < 0.05 was the significance threshold. All statistical analyses were performed with SPSS statistics (IBM, Tokyo, Japan).

## 3. Results

Participants’ characteristics are shown in Table 2. The mean (standard deviation, SD) age was 43.1 (12.8) years and 63.7% were males. Of these participants, 56.4% were married, 56.6% were educated for 12 years or more, 12.0% were in supervisory or management positions and 36.7% exercised once or more per week.

Exploratory factor analysis of the ASWS obtained the one-factor structure shown in Table 1. To show exclusive measurement of ASWS items, Table 3 presents correlations among them. No correlations were excessively high (e.g., >0.70).

Cronbach’s alpha was 0.86. Pearson’s correlations of the variables are shown in Table 4. Convergent validity was supported by findings that ASWS scores had a moderately positive correlation (*r* = 0.489) with job control, as we hypothesized (Table 4). As correlations were not excessively high (e.g., >0.70), this result also indicated that the ASWS has discriminant validity [26]. Criterion validity of the ASWS was supported as follows: Psychological distress was significantly inversely related and work engagement was significantly positively related to ASWS scores in both bivariate analyses (Table 4) and multivariate analyses adjusted for potential confounders (age, sex, marital status, educational level, work status, exercise, job control and sense of contribution) (Table 5).

**Figure 1:**
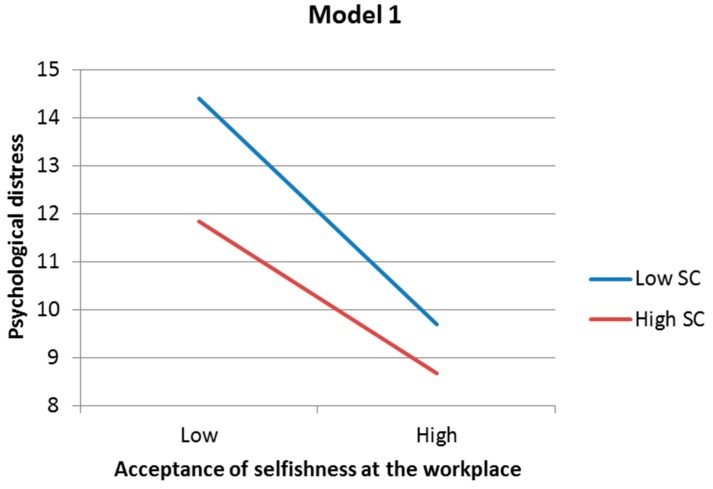

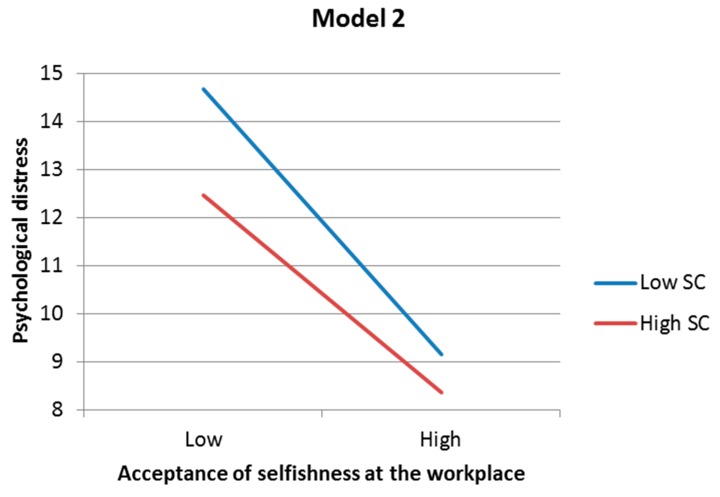
Regression lines and predicted values illustrating significant interaction effects of acceptance of selfishness at the workplace and sense of contribution on psychological distress in models 1 and 2. SC = sense of contribution.

**Figure 2:**
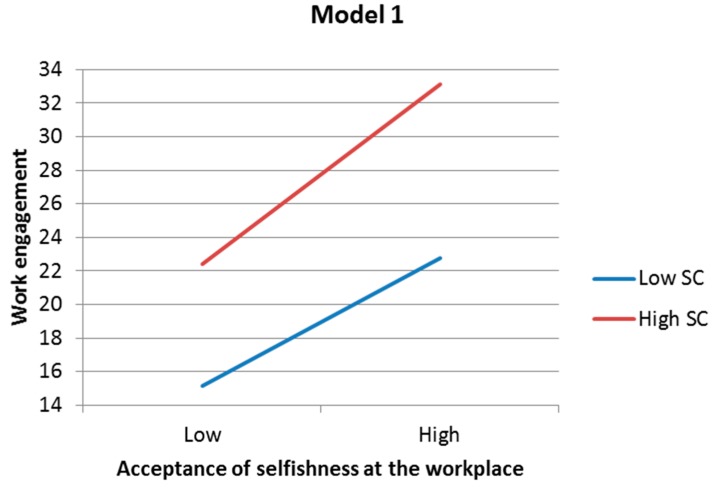

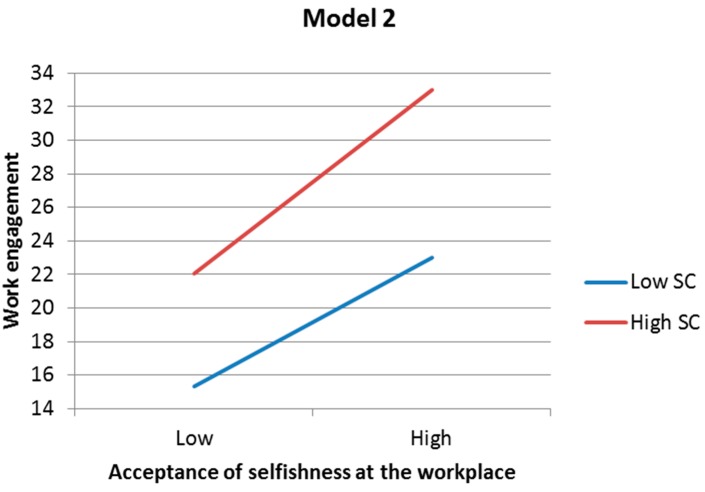
Regression lines and predicted values illustrating significant interaction effects of acceptance of selfishness at the workplace and sense of contribution on work engagement in models 1 and 2. SC = sense of contribution.

As for the second aim, acceptance of selfishness at the workplace, a sense of contribution and interaction of these two aspects were significantly and independently associated with psychological distress and work engagement in both regression models 1 and 2 (Table 6). In Figure 1, regression lines and predicted values illustrating significant interactions in both models 1 and 2 show that the higher the sense of contribution, the greater the negative association between acceptance of selfishness at the workplace and psychological distress will be (the slope of the line). When both a sense of contribution and acceptance of selfishness at the workplace are low, psychological distress becomes high. In Figure 2, regression lines and predicted values illustrating significant interactions in both models 1 and 2 show that the higher the sense of contribution, the greater the positive association between acceptance of selfishness at the workplace and work engagement will be (the slope of the line). When both a sense of contribution and acceptance of selfishness at the workplace are high, work engagement becomes high.

## 4. Discussion

The present study investigated the validity and reliability of the ASWS, a newly developed, six-item questionnaire for acceptance of selfishness at the workplace. The ASWS showed high internal consistency and acceptable convergent, discriminant and criterion validity. Results show that the ASWS is a psychometrically adequate measure.

This study also confirmed Maslow’s hypothesis of synergy. If a worker behaves selfishly at the workplace and contributes to others, he/she should be psychologically healthy [1]. Significant interactions supported the hypothesis. To our knowledge, these seem to be new findings.

Recent studies have suggested associations between workplace social capital and mental health [27,28]. However, only a few studies have suggested how to elevate workplace social capital [29,30]. An elevation of acceptance of selfishness at the workplace followed by an elevation of Maslow’s synergy may elevate workplace social capital.

This study suggested that sense of contribution and acceptance of selfishness at the workplace were independently associated with lower psychological distress and higher work engagement. Interaction between a sense of contribution and acceptance of selfishness at the workplace accelerated the associations. However, elevating a sense of contribution might be difficult because human senses and behavior may change unexpectedly; however, as mentioned before, acceptance of selfishness at the workplace depends on labor regulations, which can be changed in each department. The degree of ‘living a calling’, workplace social capital and psychological health can be compared between workers in departments that allow selfishness at the workplace and those in departments that do not. For such intervention studies, if possible, randomized controlled trials are needed.

We must also note several limitations. First, all measurements were self-reported, and therefore future studies need more objective measurements. Second, because we used convenience sampling, results might not be applicable to the entire workforce. However, since we included workers from three different organizations and obtained a response rate of more than 60%, some degree of generalizability can be expected. Third, use of a cross-sectional design did not allow us to determine causality in our results; thus, future intervention research (mentioned previously) is needed. Fourth, acceptance of selfishness at the workplace and its effects on mental health may vary depending on job types. For example, at baseline, non-manual workers (e.g., engineers, technicians, teachers, physicians, nurses, clerks, accountants, data-entry operators, sales clerks, merchandise selling professionals, real-estate salespersons, *etc.*) can have more acceptance of selfishness at the workplace than manual workers (e.g., drivers, transporters, telephone operators, tool-makers, assembly-line operators, carpenters, construction assistants, *etc.*). However, because the latter group may seldom expect elevation of acceptance of selfishness at the workplace, a smaller elevation of acceptance of selfishness at the workplace may promote mental health more greatly in the latter group than in the former. For more generalizability of our findings, acceptance of selfishness at the workplace and its effects on mental health should be investigated in various job types.

## 5. Conclusions

The ASWS, a newly developed questionnaire for the acceptance of selfishness at the workplace, is a psychometrically adequate measure. Interactions showed that when a sense of contribution and acceptance of selfishness at the workplace are low, psychological distress becomes high, while when a sense of contribution and acceptance of selfishness are high, work engagement becomes high. Maslow’s hypothesis of synergy was thus confirmed.

## Figures and Tables

**Table 1:** Exploratory factor analysis of items of the Acceptance of Selfishness at the Workplace Scale using a least squares method and promax rotation (*n* = 656).

Items	Factor Loading
1. I can question freely at the workplace.	0.65
2. I demonstrate my abilities at the workplace.	0.63
3. I can behave freely at the workplace.	0.70
4. I can say what I want to say at the workplace.	0.79
5. There is a chance to move my idea to execution at the workplace.	0.73
6. I can express myself at the workplace.	0.74
Variance explained (%)	58.5

**Table 2:** Participant characteristics (*n* = 656).

Characteristics	*n*	%	
Male	418	63.7	
Married	370	56.4	
Educated for 12 years or more	371	56.6	
Supervisory or management position	79	12.0	
Exercising once per week or more	241	36.7	
	**Mean**	**SD**	**Range**
Age (years)	43.1	12.8	19–70
ASWS ^a^ score (6–30)	20.4	4.6	6–30
Job control (24–96)	65.3	10.1	30–90
Psychological distress (6–30)	11.3	5.1	6–30
Work engagement (0–54)	23.7	12.4	0–54
Sense of contribution (7–28)	18.7	4.2	7–28

^a^ Acceptance of Selfishness at the Workplace Scale. SD = standard deviation.

**Table 3:** Correlations ^a^ of items of the Acceptance of Selfishness at the Workplace Scale (*n* = 656).

Items	Item 1	Item 2	Item 3	Item 4	Item 5	Item 6
Item 1	1					
Item 2	0.357	1				
Item 3	0.454	0.390	1			
Item 4	0.584	0.424	0.564	1		
Item 5	0.449	0.481	0.536	0.556	1	
Item 6	0.428	0.580	0.480	0.584	0.520	1

^a^ Spearman’s correlation; All *p* < 0.001.

**Table 4:** Correlations ^a^ of variables used in the ASWS ^b^ confirmation study (*n* = 656).

Measures	1	2	3	4	5
1. ASWS score	1				
2. Job control	0.489	1			
3. Psychological distress	−0.466	−0.129	1		
4. Work engagement	0.524	0.296	−0.267	1	
5. Sense of contribution	0.446	0.244	−0.345	0.521	1

^a^ Pearson’s correlation; ^b^ Acceptance of Selfishness at the Workplace Scale. All *p* < 0.001.

**Table 5:** Multivariable linear regression analyses in the ASWS ^a^ confirmation study (*n* = 656).

Independent Variable	Psychological Distress as the Dependent Variable		Work Engagement as the Dependent Variable	
	β ^b^	*p*	β	*p*
Acceptance of selfishness at the workplace	−0.48	<0.001	0.37	<0.001
Age	−0.14	0.001	0.08	0.040
Male	0.13	<0.001	−0.01	0.732
Married	0.03	0.447	−0.01	0.724
Educated for 12 years or more	0.04	0.200	0.00	0.961
Supervisory or management position	0.07	0.085	−0.08	0.028
Exercising once per week or more	0.05	0.193	0.09	0.007
Job control	0.11	0.005	0.05	0.207
Sense of contribution	−0.14	<0.001	0.34	<0.001

^a^ Acceptance of Selfishness at the Workplace Scale; **^b^** Standardized regression coefficient.

**Table 6:** Multivariable linear regression analyses including interactions in the study confirming Maslow’s hypothesis (*n* = 656).

Independent Variable	Psychological Distress as the Dependent Variable				Work Engagement as the Dependent Variable			
	Model 1		Model 2		Model 1		Model 2	
	β ^a^	*p*	β	*p*	β	*p*	β	*p*
Acceptance of selfishness at the workplace	−0.38	<0.001	−0.47	<0.001	0.37	<0.001	0.37	<0.001
Sense of contribution	−0.17	<0.001	−0.15	<0.001	0.35	<0.001	0.34	<0.001
ASW ^b^ × Sense of contribution (interaction)	0.08	0.015	0.08	0.022	0.07	0.026	0.07	0.017
Age			−0.14	0.001			0.08	0.037
Male			0.13	<0.001			−0.01	0.767
Married			0.03	0.409			−0.01	0.772
Educated for 12 years or more			0.04	0.227			0.00	0.897
Supervisory or management position			0.05	0.172			−0.09	0.011
Exercising once per week or more			0.04	0.234			0.08	0.009
Job control			0.12	0.004			0.05	0.193

^a^ Standardized regression coefficient; ^b^ Acceptance of selfishness at the workplace.

## Abbreviations

The following abbreviations are used in this manuscript:

ASWS	Acceptance of Selfishness at the Workplace Scale
JCQ	Job Content Questionnaire
K6	Kessler Psychological Distress Scale
UWES	Utrecht Work Engagement Scale
SCS	Sense of Contribution Scale
SD	Standard deviation
ASW	Acceptance of selfishness at the workplace
SC	Sense of contribution
